# Main Dietary Patterns for Healthy Aging and Well-Being

**DOI:** 10.3390/nu17122009

**Published:** 2025-06-16

**Authors:** Graziano Vinci, Federica Davì, Teresina Pellegrino, Roberta Fusco, Marika Cordaro, Rosanna Di Paola

**Affiliations:** 1 Department of Chemical, Biological, Pharmaceutical and Environmental Science, University of Messina, 98122 Messina, Italy; graziano.vinci@studenti.unime.it (G.V.); federica.davi@studenti.unime.it (F.D.); rfusco@unime.it (R.F.); 2 Department of Veterinary Sciences, University of Messina, 98168 Messina, Italy; tpellegrino@unime.it (T.P.); dipaolar@unime.it (R.D.P.); 3 Department of Biomedical and Dental Sciences and Morphofunctional Imaging, University of Messina, 98125 Messina, Italy

**Keywords:** aging, healthy aging, oxidative stress, healthy dietary patterns

## Abstract

Life expectancy around the world has increased significantly in recent decades, but chronic diseases and an aging population have also grown considerably. Accordingly, the world’s research attention has increasingly focused on the study of possible dietary patterns that can promote healthy aging and the well-being of individuals. **Method**: This review exposes three different dietary patterns that share various foods which, individually, could promote healthy aging. Following an intensive literature search, the choice of dietary patterns included the Mediterranean diet, the plant-based diet, and the Nordic diet. **Results**: The foods selected in this review, with the aim of promoting healthy aging and individual well-being, are those shared by the three diet patterns (Mediterranean diet, plant-based diet, and Nordic diet). In particular, the possible positive effects of these foods were investigated through the study of various pathways involved in the aging process, as well as oxidative stress, which underlies aging itself. **Conclusions**: Although the results are very encouraging, as they show a beneficial effect of the aliments examined, to date, many hallmarks of aging, as well as its characteristic pathways, are not concretely investigated, especially considering the foods examined. For example, deregulated nutrient sensing, stem cell exhaustion, and cellular senescence are additional important hallmarks that are not currently examined.

## 1. Introduction

Currently, innovations in technology, medicine, and biomedicine have enabled humans to increase their lifespans. For instance, 9% of the current European population is over the age of 65. It is forecast that this figure will reach 25% by the year 2050. These data are not coincidental. European populations are recognized for consuming some of the diets with aliments identified to the promote healthy aging (HA) and well-being of individuals; these diets comprise the Mediterranean diet, a plant-based diet, and the Nordic diet.

**Figure 1:**
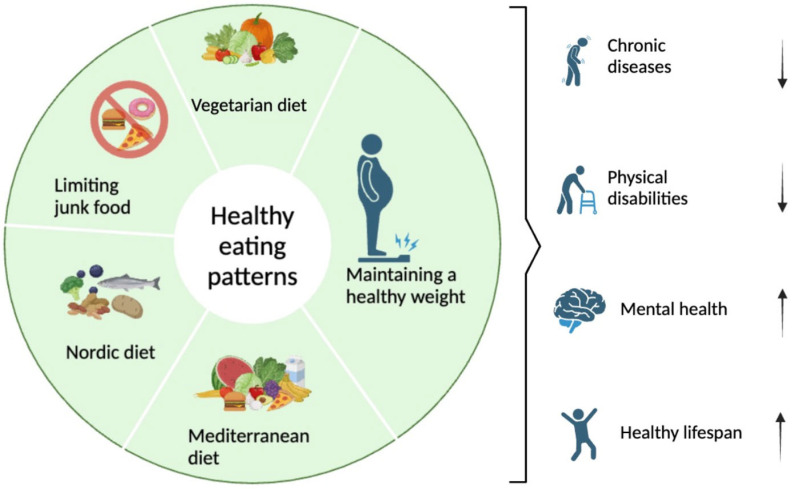
Healthy eating patterns and advice for promoting the well-being of individuals.

However, mortality and morbidity rates rise progressively with advancing age [1]. Several common conditions impacting older adults include sensory impairments like hearing loss, poor vision, cardiovascular diseases, diabetes mellitus, depression, dementia, and sarcopenia, gait and balance abnormalities, susceptibility to falls [2,3,4], and chronic obstructive pulmonary disease [5,6,7,8]. These diseases have several risk factors in common. Principal contributors are an unwholesome diet, accompanied by various health issues linked to a diet that is lacking in nutritional value, such as overweight, obesity, and metabolic syndrome, which can potentially affect approximately 75% of individuals over the age of 60 [9,10,11]. There is some evidence that elements of the diet, such as total energy and macronutrient content, play significant roles in modulating the aging process and longevity, as well as the development of age-related diseases (i.e., cardiovascular and cerebrovascular diseases, cognitive decline and dementia, and cancer) (Figure 1) [12,13,14,15]. As the proportion of older individuals continues to grow, this shift is linked to a heavier disease burden and greater expense, necessitating the implementation of evidence-based preventive policies and healthcare initiatives that promote HA to guarantee the future viability of social healthcare systems and pension plans. Healthy diet patterns with potential anti-aging properties are crucial for preventing the onset of chronic diseases and supporting a HA process [16,17]. Therefore, the aim of this review is to explore the main healthy dietary patterns associated with HA and the well-being of individual, focusing on some bioactive molecules that can contribute to this goal.

## 2. Methods

**Figure 2:**
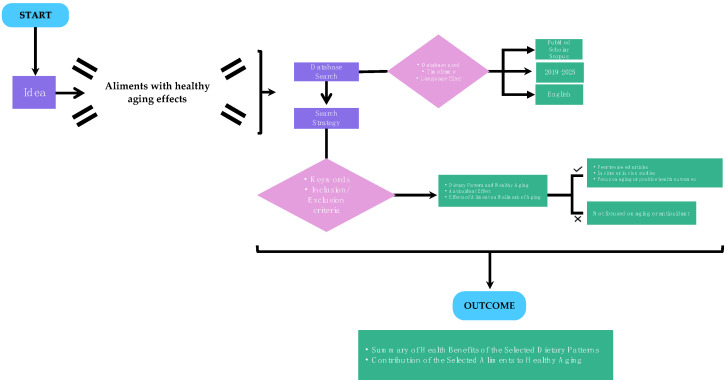
Flowchart diagram explaining the workflow of the paper.

This bibliographic review investigates the health benefits of three major dietary patterns—the Mediterranean, plant-based, and Nordic diets—with a particular emphasis on their roles in promoting healthy aging and well-being. All the research was carried out across three major databases: PubMed, Google Scholar, and Scopus, focusing on studies published between 2019 and 2025, excluding earlier research to ensure up-to-date insights. The search strategy utilized both controlled vocabulary and free-text keywords, including “aging,” “healthy aging,” and “dietary pattern and healthy aging/antioxidant effect”. These were combined with terms related to the specific diets under investigation (e.g., “olive oil against mitochondrial dysfunction” or “antioxidant effects of citrus”). In addition, (1) English-language peer-reviewed publications, (2) research with in vitro or in vivo studies, (3) studies with human subjects, and (4) research with a distinct emphasis on aging or age-related health consequences constituted the requirements for inclusion (Figure 2). The research was developed based on the foods in common among the three selected diets. Initially 17,000 articles were selected based on these conditions; most were excluded for discussing unsuitable aliments, as well as for falling outside of the selected time span (2019–2025), narrowing the selection to about 1000. An additional 800 were then removed for not being reviews. This process yielded a final selection of 135 articles for reference in this study.

## 3. Aging and Healthy Aging

**Figure 3:**
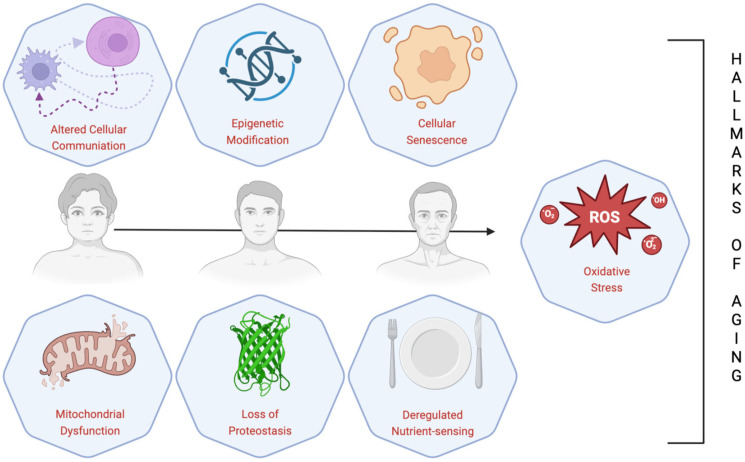
Scheme of commonly known hallmarks of aging.

Human curiosity about the aging process dates back nearly to the dawn of time. Nevertheless, its intricacy has complicated its accurate comprehension and thus, its definition. Currently, aging is defined as a progressive process associated with reduction in function and growing risk of mortality, which can be pathological and physiological. The term “pathological aging” refers to the alterations in senile aging caused by several outside variables, including cancer, degenerative joint diseases, diabetes, Parkinson’s disease, Alzheimer’s disease, cardiovascular and cerebrovascular disorders, central nervous system disorders, and the decline in several organ functions [18,19,20,21,22,23,24]. López-Otín et al. described nine cellular and molecular hallmarks of aging which are thought to set the stage for the aging process and which together establish the acknowledged physiological aging trajectory [25]. These include telomere attrition, DNA instability, mitochondrial dysfunction, cellular senescence, stem cell exhaustion, deregulated nutrient-sensing, loss of proteostasis, epigenetic changes, and altered intercellular communication (Figure 3) [25]. Recently, three more signs of aging have been added: disabled macroautophagy, chronic inflammation, and dysbiosis. In addition, these hallmarks share a common factor: oxidative stress (OS). OS occurs when there is an imbalance between the generation and removal of free radicals by the antioxidant defense system. Not surprisingly, under OS conditions, the oxidants harm different cellular components like membranes, proteins, lipids, and DNA [26]. Aging brought on by OS and its related conditions degrades tissues and upsets homeostasis [26]. Additionally, OS causes aberrant signaling in the mitochondria, which alters mitochondrial homeostasis by modulating reactive oxygen species (ROS) signaling and resulting in age-dependent cellular damage [25,26]. Scavenger enzyme activity is reduced by the progressive generation of ROS, which also promotes a pro-oxidant state that negatively influences cells, resulting in consequent cell aging [26]. However, it is erroneous to believe that aging is solely a negative process; indeed, another concept regarding aging is “healthy aging”, defined as the process by which functional abilities that promote well-being in later life are developed and maintained [27]. Even though significant advancements in science, technology, public health, and medicine have allowed older people to live longer than earlier generations, living longer is not directly associated with living healthier [28]. In this sense, research on HA conducted in recent decades has improved people’s quality of life and well-being [27].

Although studies on the distinguishing characteristics of aging have been ongoing for years, the focus on appropriate diets and the impact of bioactive molecules, contained in the diets themselves, is growing in significance. This suggests a possible involvement of healthy dietary patterns as a means to promote HA [29]. In this context, evidence present in the literature reveals that nutrients consumed individually or in consumed foods interact by influencing a set of metabolic processes [30]. Thus, suggestions for HA would have greater significance if the evidence supporting dietary patterns were examined. Although genetic factors exert the greatest effect on aging, important evidence suggests that another key role is played by environmental and lifestyle factors, e.g., a poor diet. For example, the impact of junk food on individual health serves as proof of the centrality of eating a healthy diet. The term “junk food” refers to all the foods with elevated levels of hydrogenated fats, high rates of sugar, and low levels of fiber [31]. In this context, the main junk food is represented by fast food, as well as industrially ultra-processed food [31]. Some examples of this type of food include candy, chips, cookies, French fries, chewing gum, hamburgers, hot dogs, ice cream, juice, soft drinks (especially sweetened carbonated beverages), and many packaged sweets [32]. One of the primary factors affecting quality of life and potentially contributing to the accelerated aging process is prolonged consumption of this type of food, combined with an inactive lifestyle; indeed, as visualized in Table 1 [32,33], there are many negative impacts of junk food and its components on human health. For example, childhood obesity grew quickly from 1975 to 2016, with the rate increasing from 4 to 18% [34]. These data are correlated with the high intake of junk food and fast food in general.

## 4. Main Dietary Patterns for Healthy Aging

**Figure 4:**
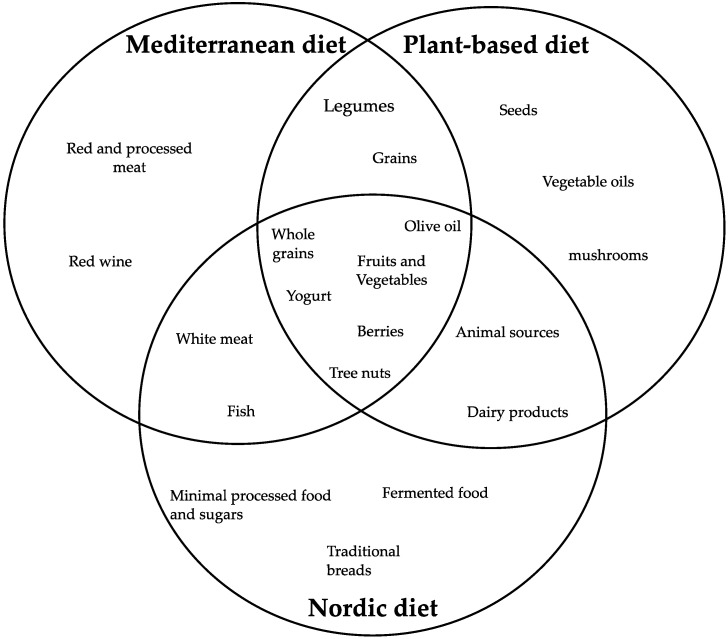
Diagram highlighting aliments in common between the Mediterranean diet, plant-based diet, and Nordic diet.

Primarily, a “dietary pattern” is a list of all the foods and drinks a person regularly consumes, with each component working in concert to influence health. As outlined in the Dietary Guidelines for Americans (DGA) 2020–2025, dietary patterns consists of nutrient-rich foods and drinks from various food class in recommended quantities and within set calorie constraints [36]. In this regard, a recommended healthy dietary pattern, which consists of appropriate macro- and micronutrients and sufficient hydration, could support the physiological cellular process. Macronutrients (i.e., carbohydrates, proteins, fats, and fiber) furnish the energy required for the cellular functions that are essential for day-to-day operations [37]. Micronutrients (i.e., vitamins and minerals) are necessary in comparatively minor amounts for normal growth, development, metabolism, and physiologic functioning [38]. Carbohydrates comprise the first fuel that the body utilizes during cellular metabolism, and they are mainly present in grains, fruits, legumes, and vegetables. Beyond providing energy, dietary proteins contain amino acids, including essential amino acids (amino acids that the body needs but is unable to self-produce). Both plant (legumes, soy products, grains, nuts, and seeds) and animal (meat, dairy, fish, and eggs) sources provide dietary proteins [39]. Lipids, also known as fats, are the main structural elements of cell membranes, with dietary fats comprised of four types, namely monounsaturated, polyunsaturated, saturated, and trans fats [40]. These various forms are typically mixed to make up a food’s fat content. Saturated fats are more prevalent in animal products (and some plant-derived oils), while unsaturated fats are present in a wide range of foods, such as nuts, seeds, fish, and many plant-derived oils [39]. Small amounts of trans fats can also be found in animal products, such as ruminant trans fats from cows, sheep, and goats, but they are mostly produced during the processing of vegetable oils [39]. Omega-3 and omega-6 fatty acids are polyunsaturated fatty acids vital for normal growth and reproduction. They are essential lipids that cannot be produced in the body and therefore, must be attained through the diet [39]. On the basis of these characteristics, certain traditional diets that present all these types of macro- and micronutrients have cultural roots and have been connected to HA. In this respect, three dietary patterns are developed, based on the cited elements; these include the Mediterranean diet, a plant-based diet, and the Nordic diet, which, as visualized in Figure 4, feature certain foods that could play a central role in HA.

## 5. Aliment and Dietary Patterns Versus Hallmarks of Aging 

### 5.1. Mediterranean Diet

The Mediterranean diet (MD), which was prevalent in nations like Greece, Italy, and Spain in the 20th century prior to the globalization of food production, processing, and distribution, is one dietary strategy that has received special attention in this respect [41]. The MD is most strongly linked to traditional Mediterranean olive-growing regions and has long been associated with long life expectancy and low rates of chronic illnesses [42]. The MD is mainly composed of low to moderate intake of poultry, fish, red meat, and wine in restraint. Also, it consists of an elevated consumption of plant foods, i.e., fruits, vegetables, breads and other cereals (generally from whole grains), potatoes, legumes, and tree nuts, with sweets made with sugars or honey a few times a week. Although the MD is distinct from other healthy diet patterns due to its relatively high consumption of nuts, olive oil, and modest amounts of wine, especially red wine, during meals, it can be regarded as a diet that is predominantly plant-based [42].

The Mediterranean area is known for receiving a significant amount of sunlight; accordingly, many of the region’s distinctive fruits, like grapes and olives, have developed several compounds, such as phenols, that guard against oxidative damage brought on by the stress caused by extended exposure to UV light. Since this area is renowned for its lower prevalence of specific diseases, this distinct feature of the fruit’s growth and evolution become significant [43].

### 5.2. Effects of Olive Oil Against Aging

In this respect, olive oil is an important component of MD due to is bioactive compounds, particularly phenols (i.e., hydroxytyrosol, tyrosol, oleocanthal, and oleuropein). Despite making up a small portion of its composition, these compounds are essential because of their biological, anti-inflammatory, and antioxidant qualities, which interact with intricate molecular mechanisms to control inflammatory OS responses, delaying the aging processes of cells [44,45]. For instance, hydroxytyrosol is a well-studied polyphenol compound characterized by a benzene ring with two hydroxyl groups and an ethanol group, making it particularly reactive against free radicals, granting it powerful antioxidant properties [46]. According to recent studies, hydroxytyrosol can pass through cell membranes, shielding brain tissue and presenting diseases like Alzheimer’s [47]. Its potent antioxidant properties aid in preventing DNA damage and lipid peroxidation [48]. Tyrosol maintains the integrity of the cell wall and lowers the risk of lipid peroxidation, which greatly increases cardiovascular protection [46]. This increases RBC resilience in stressful situations and slows down cellular aging. Oleocanthal works by blocking the cyclooxygenase enzymes (COX-1 and COX-2), which lowers the synthesis of inflammatory molecules [49]. Lastly, oleuropein is renowned for its potent antioxidant, anti-inflammatory, and cardioprotective effects [50]. Additionally, oleuropein has been demonstrated to induce apoptosis in cancer cells, increasing its potential as a preventative and protective agent for general health [51]. In addition to being a characteristic of aging, mitochondrial dysfunction is becoming more widely acknowledged as one of the initial occurrences in Alzheimer’s disease. Specifically, cell and animal models of Alzheimer’s disease have shown decreased mitochondrial biogenesis, ATP production, and oxygen consumption [52]. In this context, the study conducted by Grewal et al. [53] explored the possible alteration of the mitochondria, with a focus on the ATP levels, which play a key role in mitochondrial dysfunction. Linked to the above findings, the authors have chosen olive oil because it could enhance mitochondrial respiration by intensifying the levels of ATP, thanks to its bioactive compounds (particularly polyphenols) [53]. In addition, it was evaluated as one important marker of mitochondrial content, i.e., citrate synthase activity [54]. In this respect, there was an increase in citrate synthase activity in SH-SY5Y-APP695 cells as a result of treatment with olive oil. Given that mitochondrial biogenesis is known to be compromised in Alzheimer disease and especially in aging, this may be a sign of a positive effect [55]. Additionally, the authors have investigated the expression of various genes implicated in mitochondrial biogenesis. An important transcriptional factor in this context is peroxisome-proliferator gamma coactivator 1-α (PGC-1α), which is activated via deacetylation by sirtuin 1 (SIRT1) or phosphorylation via AMP-activated protein kinase (AMPK). PGC-1α also activates other factors such as nuclear-respiration factor 1 (NRF1) and mitochondrial transcription factor (TFAM), which play an important role in mitochondrial biogenesis [56,57]. Surprisingly, olive oil compounds have enhanced the expression of these genes. These results suggest that olive oil and its compounds (in particular, polyphenols) could have a positive influence on the mitochondrial bioenergetic system. Therefore, these findings could demonstrate how olive oil improves cellular energy metabolism and likely prevents mitochondrial dysfunction.

Another study conducted by Mercedes Blanco-Benítez et al. aimed to probe the possible antioxidant and anti-inflammatory effects of olive oil noteworthy in mitochondrial dysfunction [58]. Study findings support that olive oil may enhance the expression of transcription factor A (TFAM) in the mitochondria, boost the number of copies of mitochondrial DNA (mtDNA), and increase the mass of the mitochondria. Olive oil also encourages the de novo synthesis of the mitochondria and their capacity to scavenge the ROS produced by them. Additionally, olive oil increases the SIRT1 protein through the consequent activation of PPARγ and PGC1-α. As described previously, PGC1-α is linked to the biogenesis of the mitochondria. When it is upregulated, mitochondrial function is enhanced, which raises COX activity [58].

### 5.3. Effects of Avocado Against Aging

The Mediterranean population consumes elevated quantities of fruits and vegetables. In this context, currently, the highest consumed and imported fruit in this region is the avocado [59]. In addition, data suggest that the Mediterranean area has expanded fruit cultivation to include avocados. Indeed, as stated in the latest OPM statistics, in Spain, avocado cultivation covers over 12,832 hectares (ha), with most of this area located in SE Spain (Andalucía), at 10,594 ha (83%), followed by the Canary Islands (13%), and the Valencia provinces (9%) [60].

The many bioactive compounds found in avocado oil (AO) have recently attracted a significant amount of attention because they may have positive anti-aging effects. These compounds appear to directly regulate the production and activity of antioxidant enzymes, and avocado oil’s ability to suppress pro-inflammatory cytokines while boosting anti-inflammatory proteins might explain its anti-inflammatory properties [61]. Moreover, it was discovered that avocado oil is an innate activator of sirtuin 1 (SIRT1) [62]. By virtue of its neuroprotective nature, SIRT1 has been declared to be a protein involved in fighting age-associated neurological alteration [63]. The oleic acid-derived oleoyl ethanolamide (OEA), a naturally occurring substance in the human body whose levels are impacted by dietary oleic acid intake, is linked to the anti-aging effect of avocado oil. Therein, the oleic acid acts as an endogenous ligand of the nuclear receptor protein that functions as a transcription factor, i.e., peroxisome proliferator-activated receptor alpha (PPAR-α). Given its effect of improving insulin sensitivity, it has been proposed as a possible target for both the treatment of obesity and aging markers, thanks to its anti-inflammatory and antioxidant properties [62]. Also, AO compounds could suppress iron-overload-induced harm, which correlates with a possible phenomenon that could accelerate cell senescence; indeed, redox imbalances cause ferroptosis, which is linked to aging, as well as its related morbidity and increased mortality [64]. Furthermore, in an iron overload model in mice, AO compounds were found to decrease liver damage and inhibit liver lipid peroxidation [65]. Avocado oil presents an important range of polyunsaturated fatty acids (PUFAs), affirmed by a study that reported that linoleic acid, at 48.77%, comprises its main PUFA [66]. These compounds of AO have shown the capacity to control inflammatory reactions in the central nervous system, specifically in the microglia, the main mediators of inflammation associated with aging [67,68].

Another study conducted by Motta, J.R. et al. evaluates the helpful effect of avocado oil against OS and one more possible aging hallmark, apoptosis [69]. In this research, the authors exposed the SH-SY5Y cells to hydrocortisone (HC), the active cortisol molecule. As previous studies explain, exposure to HC could have a cytotoxic effect on SH-SY5Y cells, and the findings supported this theory, indicating that undifferentiated neuroblastoma cells (SH-SY5Y) could be used as an in vitro HC-cytotoxic model [70,71]. In fact, the results showed that HC caused mortality to increase beginning at a concentration of ≥0.3 ng/mL, likely causing OS and apoptotic events [69]. Specifically, elevated levels of the CASP-3 protein suggest that prolonged exposure to cortisol causes significant dysfunctions that are linked to the aging of the brain [72]. For instance, oxidative damage and other harmful triggers frequently converge at CASP-3 activation in the aging brain [72]. Increased levels of macromolecule oxidation (oxDNA, LPX, and PCAR) were seen in HC-exposed cells, which could contribute to apoptotic activation. Against these cytotoxic effects, avocado oil reverses the rates of mortality brought on by HC exposure and reduces OS and apoptosis [69].

## 6. Plant-Based Dietary Pattern

Another dietary pattern that shares some aliments with the previous diet is the plant-based dietary pattern. Plants and their constituent elements, such as vitamins, flavonoids, and carotenoids, are known to possess antioxidant potential that could aid in the prevention and treatment of chronic diseases brought on by ROS [73]. Furthermore, it has been shown that eating more plant-based foods is one of the main lifestyle changes resulting in the prevention of age-related illnesses and the maintenance of general health [74,75]. Furthermore, numerous studies have demonstrated a favorable association between plant-based diets and (PBS) and successful or HA [76,77].

### 6.1. Effects of Fruits Against Aging

As explained previously, one constituent of this type of dietary pattern is fruits; for example, berries are predominantly consumed by people who adopt PBS. In this context, Fan H. et al. focused on alcoholic fatty liver disease (ALFD), characterized by excess triglyceride enhancement in the liver [78]. Even in this type of illness, OS and mitochondrial dysfunctions are connected due to their pivotal role in pathogenesis [78,79]. ROS buildup drives damage to mitochondrial DNA (mtDNA) and initiates extensive mtDNA deletions. In a vicious cycle, persistent DNA damage and depletion cause mitochondrial dysfunction and speed up the production of ROS [80]. In this study, the authors selected blueberries as a possible treatment to counter OS and mitochondrial damage. In addition, they combined blueberries and probiotics, since blueberries encourage the development of probiotic bacteria, which raise blueberries’ biological activity and may promote a synergistic effect [81]. The authors discovered that in an ALFD model, blueberries and probiotics enhanced the functionality of the liver mitochondria, lowering mitochondrial OS by blocking malondialdehyde and ROS and triggering glutathione (GSH) and SOD. Thus, by controlling mitochondrial function, blueberries and probiotics significantly reversed the pathophysiology of alcohol-induced ALFD. As in previously, the authors focus on SIRT1, and they found that the livers of ALFD mice displayed significantly lower levels of SIRT1 mRNA and protein than did the livers of normal mice. They also found that SIRT1 deficiency significantly decreased the capacity of blueberries and probiotics to defend the mitochondria and fight OS, suggesting that blueberries and probiotics inhibit OS and mitochondrial function through SIRT1 [78]. SIRT1 silencing inhibited PGC-1α expression and reversed the effect of blueberries and probiotics, whereas PGC-1α expression increased significantly following the blueberry and probiotic intervention. This suggests that the SIRT1 activation of PGC-1α was required for the regulation of the hepatocyte mitochondria via blueberries and probiotics in ALFD [78]. Blueberries and probiotics work in concert to hinder ALFD by lowering OS, enhancing mitochondrial function, and preserving mitochondrial structure. According to these findings, blueberries and probiotics could be a promising dietary supplement to prevent ALFD and may even have anti-aging properties regarding the molecular mechanisms of ALFD [78]. During the explanation of the various aging hallmarks, the list also includes telomere attrition and of course, telomere length (TeLe). Although it is believed to be stable from childhood through young adulthood, this phenomenon is highly variable among individuals and starts to decline in older adulthood [82]. Reduced life expectancy and higher rates of age-related syndrome development are connected with shorter telomeres [83]. Indeed, cellular senescence and aging have been linked to telomere shortening and damage. However, studies and scientific research have demonstrated that with some lifestyle approaches, for example, the adoption of healthy dietary patterns, this complication could be altered [84]. Indeed, to support this argument, in recent years, numerous studies have paid attention to five populations living in specific areas of the Earth, labeled as “blue zones”, due to their unusual longevity.

The “blue zones” comprise Sardinia (Italy), Okinawa (Japan), Loma Linda (California), Ikaria (Greece), and the Nicoya Peninsula (Costa Rica), where the main sources of nutrition of these populations are plant-based [85,86]. The study of Gong, Y. et al. focused on this argument [87]. The authors selected four different dietary patterns, each with several characteristics, for example, the “high energy density” pattern (characterized by sugary drinks, fried foods, and wheat flour), the “traditional” pattern (including red meat, rice, and pickled vegetables), the “macho” dietary pattern (consisting mainly of animal foods and alcohol), and the “vegetable-rich” diet. Out of the four dietary patterns identified in the study, only the latter, characterized by a higher intake of fruits, whole grains, various vegetable groups, dairy products, nuts, eggs, and tea, was positively connected with TeLe in women. However, no significant difference in TeLe was observed in men [87]. An interesting study by Xiude Li et al. focused attention not only on a plant-based diet but also on investigating the healthy or unhealthy effects correlated with the types of foods selected [88]. The primary focus of the author’s thesis was to explore the reality that not all plant-based foods are regarded as healthful. To address these limitations, they utilize the three Satija dietary indices: the overall plant-based diet index (PDI), the healthy PDI (hPDI), and the unhealthy PDI (uPDI) [89]. According to earlier studies, uPDI has high correlations with aging and mortality, while hPDI has an inverse relationship with both [90,91,92]. The proof of this study suggests that, on a biological level, a plant-based diet high in nutritious plant foods is linked to HA, while a plant-based diet high in unhealthy plant foods may have the opposite negative effects [88]. Moreover, the healthy plant-based diet has several dietary elements in common with MD, consisting of consuming fewer red and processed meats and more whole grains, fruits, vegetables, legumes, and nuts. In this sense, the relevance of this study could also extend to the MD [92]. Therefore, vegetables, fruits, nuts, legumes, and whole grains, present in the hPDI, are sources of polyphenols and/or fiber and thus, have reportedly been linked to longer telomere length [93,94]. Conversely, some of the foods included in the uPDI, like refined grains, sugar-sweetened beverages, and sweets, are linked to OS and inflammation, which may shorten telomeres. Finally, the results point to a possible correlation between longer telomeres and the hPDI, while telomere shortening may be connected to the uPDI [88].

### 6.2. Effects of Dark Chocolate Against Aging

Another study focuses on different food that is commonly consumed by individuals who adhere to a plant-based diet: dark chocolate. Chen Li et al. have scrutinized the possible anti-aging effect of dark chocolate, based on TeLe [95]. The authors selected dark chocolate, instead of other types of chocolate, because of is larger cocoa content. Cocoa is described as antioxidant food, due to its natural bioactive substances (like flavonoids), which may prevent DNA damage and improve the nuclear integrity of cells [96]. Dark chocolate presents more cocoa content. Indeed, thanks to this, it can positively modulate TeLe by enhancing endothelial nitric oxide (NO)-synthase activity through the insulin-mediated signaling pathway, whereas the suppression of NO synthase accelerates the TeLe [97]. In addition, dark chocolate can also inhibit angiotensin-converting enzyme (ACE) activity, which indicates that TeLe is negatively impacted by the ACE deletion genotype [98]. The authors also examined the consequences of dark chocolate on the ApoA1/HDL pathway. They positively proved that dark chocolate exerts a beneficial effect on ApoA1, ApoA1/HDL and LTL in adolescents; thus, thanks to these findings, the authors promote the association between chocolate consumption and TeLe [95]. Finally, the authors conclude that the positive effects of dark chocolate on TeLe could be precisely associated with the ApoA1/HDL pathway; therefore, they promote the consumption of two or more servings/week of dark chocolate because of its positive effects on TeLe compared to the results for those who did not consume this aliment [95].

### 6.3. Effects of Yogurt Against Aging

Another main aliment of the cited diet is yogurt and similar foods. This type of food contains lactic acid bacteria, which make up a class of probiotics with countless healthy effects. Indeed, besides lactic acid bacteria, the fermentation process initiated by the bacteria can introduce different antioxidative peptides that may have positive effects [99,100]. Among the various beneficial effects, those of note in the present review include anti-inflammatory, antioxidant, and anti-aging effects [101]. In this context, Shan et al. [102] evaluated the possible anti-aging effects of yogurt in relation to TeLe. In particular, the authors utilized an aging model of D-galactose in male mice. Rodents with abnormally high D-galactose levels would accumulate more ROS, which would shorten the telomeres and damage the structure of the cells [102,103]. Furthermore, they also utilized Hep52 cells. Their findings show that yogurt significantly reduced hepatic and leukocyte telomere attrition when compared to the results for the aging model. In particular, the authors evaluated senescence-associated β-galactosidase activity (as a possible marker of cellular aging and TeLe) [104]. In aging mice supplemented with yogurt and the metabolites of lactic acid bacteria (*S. thermophilus* and *L. rhamnosus*), β-galactosidase activities were significantly lower than in those observed in the aging model, but the decrease observed in the milk (control) group was not statistically significant. This means that the senescence of the aging mice was suppressed by yogurt and dairy-fermenting bacteria. In liver, heart, and brain tissues, the SOD activities were higher, while the malondialdehyde levels in the liver and heart were significantly lower than those in the aging model. These findings were attained with the supplementation of yogurt and lactic acid bacteria. Thus, the results demonstrated that yogurt and lactic acid bacteria were able to reduce senescence and increase antioxidative potential in aging mice. Consequently, they can act as TeLe modulators [102,105].

Finally, the authors also examined the consequences of these foods and metabolites to substantiate the positive effects found in vivo and even in vitro. In accordance with previous results, the effects of yogurt and lactic acid bacteria metabolites significantly reduced senescent cell numbers. They also increased TeLe. Once again, positive effects were found for OS assessment. Indeed, yogurt and lactic acid bacteria increased SOD activity; similar results were found in regards to CAT activity [102]. These possible positive effects are related to the fermentation process of probiotics (LAB). In support of this, the authors referenced a study by Hor et al. that found that cell-free culture supernatants from various lactic acid bacteria decreased telomere attrition and senescence in elderly rats [106].

## 7. Nordic Dietary Pattern

The last proposed dietary pattern is the Nordic diet. As with the previous examples, this diet is also characterized by food items with positive nutritional profiles [107]. Of course, this type of diet is built on the main resources from northern areas; for example, this is an area where plant foods like berries, cabbages, apples, pears, root vegetables, oats, whole grains, and rye thrive. These areas also present long coastlines, providing a rich source of fish [108,109].

### 7.1. Effects of Whole Grains Against Aging

As emerged from this review, whole grains are commonly consumed in all the cited diets, and certainly, these are also consumed in the Nordic diet. Actually, the potential healthful effects of whole grains are also associated with anti-aging properties. In this sense, Sooji S. et al. analyzed the possible antioxidant effects of oats (*Avena sativa*) extract in an OS model of human keratinocytes [110]. In this context, the results demonstrated that oat extract positively affects cell viability, protecting cells from cell death induced by hydrogen peroxide, and surprisingly, the lowest concentration of oat extract was sufficient to maintain cell viability. Additionally, they further investigated whether oat extract exhibits ROS scavenging properties. In fact, they discovered that oats restored the ROS intracellular contents to control levels. The study also evaluated the ability of oats to counteract OS-induced DNA damage. In this case, the authors utilized specific molecules as DNA damage markers. OS caused a rise in the phosphorylation of checkpoint kinase 1 and checkpoint kinase 2, mediators of DNA damage. Oat extract ameliorates the OS damage to DNA checkpoint molecules, with significant reductions in checkpoint kinase 1 and checkpoint kinase 2. Additionally, the overactivation of checkpoint kinase 2 leads to the downstream activation of p53, while this condition was significantly decreased by oat treatment [111]. Currently, another DNA damage marker observed to correlate with early aging is the phosphorylation of the Ser-139 residue of the histone variant H2AX [112]. The oat extract treatment in the present study significantly reduced the elevated activation, whereas OS substantially increased the phosphorylation of H2AX. Lastly, to investigate the complete antioxidant capacity of oat extract treatment, several markers of cellular apoptotic death were examined. First, the authors examined caspase-3, and oat extract treatment ameliorated its activity in regards to OS damage. Then, the modifications of the apoptotic genes, cleaved caspase-3 and cleaved caspase-7, were examined. In this case, oat extract treatment dramatically reduced the elevated levels provoked by OS in a dose-dependent manner. The cleaved poly (ADP-ribose) polymerase has also been examined because caspases mediate the activation of poly (ADP-ribose) polymerase, which results in apoptosis [113,114]. Even in this case, the dose-dependent oat extract treatment restored the cleaved poly (ADP-ribose) polymerase level.

Another coarse cereal that is shared in the cited dietary patterns is quinoa (Chenopodium quinoa Willd). This cereal, by virtue of its possible anti-aging and antioxidant properties, has received significant attention from scientists [115]. Indeed, compared to conventional cereals such as rice and wheat, quinoa contains elevated healthy ingredients, like quality protein, fiber, minerals, and vitamins [116]. To evaluate the possible health effects of quinoa, Hu Y. et al. investigated the anti-aging effects of quinoa in D-galactose-induced-aging mice models [117].

The administration of D-galactose resulted in an increase in all OS markers, as evidenced by a decrease in high-density lipoprotein cholesterol (HDL-C), SOD, CAT, and GSH-Px levels; it also induced learning and memory impairment and an increase in total cholesterol (TC), triglycerides (TG), and low-density lipoprotein-cholesterol (LDL-C) levels; the production of malondialdehyde; and an upregulation of monoamine oxidase activity in the brain. Surprisingly, the treatment with quinoa protected against OS induced by D-galactose, increasing the maturity of connective tissues, lowering the amounts of lipid peroxidation products, and increasing the activity of antioxidant enzymes [117]. These anti-aging effects obtained with quinoa administration are still unclear. Of course, the main positive effects are owed to the presence of the above cited natural polysaccharides. Therefore, to gain a deeper comprehension of the underlying mechanisms, the authors mainly investigated the forkhead box O transcription factors (FOXOs) and the p53 pathway as markers of aging. Data presented in the literature suggest that FOXOs are important transcription family factors associated with longevity in mammalian organisms because of their involvement in cellular homeostasis, and consequently, they promote HA [118,119]. In this context, a key player in the control of cell growth, proliferation, and apoptosis is FoxO3a, a member of the FOXO transcription factor family. In addition, FoxO3a interacts with p53, another crucial transcription factor. By increasing FoxO3a’s phosphorylation level, p53 activation can suppress FoxO3a’s transcriptional activity [117]. In contrast with the data, which shows an increase in p-53 levels and consequently, a decrease FoxO3a, quinoa treatment could restore these indexes. The study suggested that quinoa’s anti-aging properties may be linked to halting ROS-induced cell damage [117].

### 7.2. Effects of Tree Nuts Against Aging

Another main food of the Nordic diet, which is also shared with previous dietary patterns, is tree nuts (cashew, peanuts, etc.). In this context, the study by D’Amico R. et al. drew attention to an important metabolic disorder, hyperhomocysteinemia (HHcy) [120]. HHcy is an abnormality of methionine metabolism that can cause different cellular disorders, resulting in altered well-being and increased cellular senescence. As previously discussed, OS plays a pivotal role in the pathophysiology of HHcy damage. Therefore, the authors investigated the possible antioxidative effects of cashews in an experimental model of HHCy in rats induced via L-methionine [120]. Clinical biochemical alterations, oxidative and nitrosative stress, decreased antioxidant enzyme levels, lipid peroxidation, proinflammatory cytokine release, histological tissue damage, fibrosis, and apoptosis in the kidney, colon, and liver, respectively, were all reversed in HHcy rats by oral cashew nut administration. The authors proved that HHcy can exacerbate the alteration, caused by OS, of mitochondrial dysfunction. In this context, via numerous stimulations, the authors explain that Nrf2 is the main transcriptional activator of the HO-1 gene. This pathway, when active, can protect cells from OS [121]. During HHcy, the activity of Nrf2-HO-1 mRNA and protein expression are strongly limited [122]. However, the presence of antioxidants can lead to Nrf2 activation, which promotes the Nrf2-HO-1 pathway itself [123,124]. Thus, they found that cashew nut treatment was associated with higher serum levels of SOD, GSH, and CAT, as well as Nrf2 nuclear translocation, Nrf2, and regulated factors like HO-1 expression in all tissues. In addition, since several studies suggest that HHcy can lead to enhanced apoptosis or necrosis, the authors evaluated the correlation between HHcy, OS, and apoptosis [120,125]. Indeed, they discovered that numerous pro-apoptotic markers were increased, like Bax, p53, and caspase-3, suggesting a connection between NF-kB activation, which controls the release of these pro-inflammatory molecules, and HHcy-induced cell damage [126,127]. The results show that cashew treatment reduces NF-KB activation and consequently, the apoptotic pathways [120]. By increasing antioxidant capacity and regulating ROS-induced signaling, such as nuclear NRF-2 or NF-κB, cashew nuts demonstrated the ability to decrease tissue inflammation and OS, consequently enhancing anti-aging effects through dietary intake.

## 8. Dietary Supplements from Diets

As emerged above, all the aliments presented here are foods that are commonly consumed in the dietary patterns. However, currently, the focus on waste products from aliments is strongly increased. In this regard, nutraceuticals from these sources could comprise a powerful untapped source of health-promoting compounds that can revolutionize modern nutrition and disease prevention (with specific healthy aging effects). In addition, they could contribute to environmental sustainability through the utilization of parts of foods that are typically excluded.

### 8.1. Effects of Citrus Peels Against Aging

As Denham Harman discussed for the first time in 1972 in his “free radical theory of aging”, OS is the main cause of the alteration of macromolecules, with the consequent alteration of cellular homeostasis. This could explain the alterations in cell functions during natural aging [128]. As described previously, Mediterranean individuals consume elevated quantities of fruits and vegetables. In this vein, citrus fruits are one of the major types consumed. Despite many studies published on the possible antioxidant effects of citrus, the focus on citrus peels is poorly investigated. Novel studies advise that citrus peels could be utilized to obtain essential oils, using them for possible nutraceutical purposes given their renowned antioxidant properties. The scientific study directed by Remigante et al. aimed to investigate the possible antioxidant effects of bergamot peel and juice extract on the molecular mechanisms underlying natural aging in red blood cells (RBCs) [129]. The authors explain that RBCs are a perfect vehicle for studying OS; indeed, these cells are constantly exposed to ROS. As markers of OS, the authors investigate the levels of thiobarbituric acid reactive substances and sulfhydryl groups, which indicate OS, in membrane lipids and proteins, respectively. Bergamot extract ameliorates either the thiobarbituric acid reactive substance level increases in RBCs or the oxidation of the membrane sulfhydryl groups. These results show that bergamot peel or juice extract could successfully prevent oxidation of the RBC plasma membrane’s lipid and protein components. Furthermore, research also studied the functionality of the band 3 protein, the most expressed membrane protein in human RBC. By assessing the functioning of this protein, the authors were able to understand the possible effect of bergamot extract on RBC physiology. According to the data, the authors discovered that both extracts have a comparable protective effect on anionic exchange and may thus be crucial in reversing the functional alterations in human RBCs brought on by OS. In the end, they investigated the activity of endogenous antioxidant enzymes catalase (CAT) and superoxide dismutase (SOD). These antioxidant enzymes are crucial for human RBC and have exceptional free radical scavenging abilities. During OS events, the activities of these antioxidant enzymes are higher than those in the control cells. Therefore, the findings highlight that extract of bergamot might counteract OS in RBCs and preserve cell integrity, enhancing the endogenous antioxidant system [129].

### 8.2. Peanut Peel Extract Against Aging

The study by Annayara C.F. Fernandes et al. focuses on another type of nut, the peanut [130]. Here, the authors evaluate the antioxidant effect of peanut peel extract, an agro-industry waste, on macrophage cells, RAW264.7, in which OS was induced. The authors provide a justification for their decision to investigate the effects of this peanut extract on macrophages [130]. Macrophages are primarily involved in inflammatory responses; that is, they generate pro-inflammatory factors, cytokines, and ROS when they are stimulated. When lipopolysaccharide (LPS) stimulates RAW 264.7 macrophages, the TLR4 receptor is activated [130]. Through its interaction with LPS, TLR4 phosphorylates the MAPK signaling pathways ERK, JNK, and p38. This activates c-Jun N-terminal kinase (JNK), which in turn activates iNOS, raising NO levels. Additionally, the NF-κB pathway, which regulates pro-inflammatory enzymes like iNOS and various cytokines like TNF-α and IL-6, is activated when MAPK is phosphorylated [131]. The peanut extract inhibits the pro-inflammatory mediating compounds. Instead, peanut extract decreases NO concentrations in a dose-dependent manner. In this case, the authors explain that this result corroborates other results presented in the scientific literature, which showed the reduced production of NO in RAW264.7 cells after the administration of elderberry extract in a dose-dependent manner [132,133]. Furthermore, peanut extract has been associated with the suppression of NF-κB’s binding to the iNOS promoter, which sequentially prevents iNOS and NF-κB expression. Therefore, this extract has been revealed as a possible source of 18 flavonoids that demonstrated the extract’s encouraging capacity to subdue ROS production, lower inflammatory biomarkers, and reduce adipogenesis and lipid accumulation in vitro, with effects that exhibited dose-dependent behavior. As stated in this study, there are several possibility for using agro-industrial waste to create possible nutraceuticals with antioxidant and anti-inflammatory capabilities [130].

## 9. Strengths and Limitations

This review aims to evaluate the results of recent research (between 2019 and 2025) in the scientific literature regarding the antioxidant and anti-aging capabilities of certain foods that are part of three diets recognized to have beneficial effects on the body. Overall, these dietary patterns (Mediterranean diet, plant-based diet, and Nordic diet) emphasize the importance of food quality, variety, and preparation methods to promote long-term health and well-being.

In addition, there are relatively few current research reviews focusing on aging and food-related pathways, which provided the idea for the present work. However, the main limitations found during the intensive literature search period relate to the lack of recent studies on other types of foods and their effects on the hallmarks of aging. Still, many hallmarks of aging are currently under-investigated, and this is the main limitation of the review itself.

## 10. Conclusions

In conclusion, as initially described, unhealthy habits (e.g., junk food consumption, a sedentary lifestyle) can accelerate aging. Thus, this work aimed to analyze possible pathways implicated in the characteristics of aging and to identify foods that can counteract or limit the onset of poor aging and promote HA and individual well-being. Thus, this review investigated the foods shared between three diets, the MD, plant-based, and Nordic diet, which are commonly recognized as diets conducive to HA and which include numerous foods that possess interesting anti-aging properties. Specifically, these diets, and obviously their aliments, exhibit protection against the hallmarks of aging (e.g., mitochondrial dysfunction, telomere length), as well as antioxidant and anti-inflammatory effects. In addition, food waste (e.g., peanut peel or bergamot peel and juice) was also considered in the review; the idea is to focus on these food industry wastes because they could be exploited as nutraceuticals to improve human well-being. However, as emerged during the research, to date, there are still few studies involving the other hallmarks of aging (like deregulated nutrient sensing), nor is there much research regarding other possible foods with anti-aging characteristics. Consequently, continuing to search for possible natural solutions to improve aging and well-being is essential to avoid or postpone the insurgence of age-related diseases. In addition, with the advancement of more practical and comprehensive quality control guidelines to ensure the safety and efficacy of natural product-based therapies, i.e., nutraceuticals, this therapy might be important in promoting the HA and well-being of most individuals.

## Figures and Tables

**Table 1:** List of junk foods and the health impacts of their constituents [33,35].

Junk Food Type	Components	Health Impacts
Sweetened soda, soft drinks	High-fructose corn syrup	Weight gain and diabetes, hypertension, atherosclerosis, coronary heart disease.
French fries, ice-cream	Trans fats	Increase in inflammatory markers, cancer, and diabetes.
Burgers and sandwiches	Per/poly fluoroalkyl substances (PFAS)	Breast cancer, infertility, weakened immune system.
Processed cheese, chicken nuggets	Phosphate additives	Kidney disease, bone problems.
Processed red meats	Sodium nitrite	Stomach cancer, renal inflammation.
Candy	Sugar, sweeteners, corn syrup	Weight gain and cardiovascular health.
